# Liquid Chromatography/Tandem Mass Spectrometry Analysis of *Sophora flavescens* Aiton and Protective Effects against Alcohol-Induced Liver Injury and Oxidative Stress in Mice

**DOI:** 10.3390/antiox13050541

**Published:** 2024-04-28

**Authors:** Ye Jin Yang, Min Jung Kim, Ju-Hye Yang, Ji Woong Heo, Hun Hwan Kim, Woo H. Kim, Gon Sup Kim, Hu-Jang Lee, Young Woo Kim, Kwang Youn Kim, Kwang Il Park

**Affiliations:** 1 Departments of Veterinary Medicine, Gyeongsang National University, Jinju 52828, Republic of Korea; yang93810@gnu.ac.kr (Y.J.Y.); minjung0102@gnu.ac.kr (M.J.K.); hujiw7806@gnu.ac.kr (J.W.H.); hunskim@gnu.ac.kr (H.H.K.); woohyun.kim@gnu.ac.kr (W.H.K.); gonskim@gnu.ac.kr (G.S.K.); hujang@gnu.ac.kr (H.-J.L.); 2 Korean Medicine (KM) Application Center, Korea Institute of Oriental Medicine, Daegu 41062, Republic of Korea; jjuhye@kiom.re.kr; 3 School of Korean Medicine, Dongguk University, Gyeongju 38066, Republic of Korea; ywk@dongguk.ac.kr

**Keywords:** *Sophora flavescens* Aiton, antioxidant effects, alcohol, liver damage

## Abstract

In this study, we investigated the hepatoprotective effects of an ethanol extract of *Sophora flavescens* Aiton (ESF) on an alcohol-induced liver disease mouse model. Alcoholic liver disease (ALD) was caused by the administration of ethanol to male C57/BL6 mice who were given a Lieber−DeCarli liquid diet, including ethanol. The alcoholic fatty liver disease mice were orally administered ESF (100 and 200 mg/kg bw/day) or silymarin (50 mg/kg bw/day), which served as a positive control every day for 16 days. The findings suggest that ESF enhances hepatoprotective benefits by significantly decreasing serum levels of aspartate transaminase (AST) and alanine transaminase (ALT), markers for liver injury. Furthermore, ESF alleviated the accumulation of triglyceride (TG) and total cholesterol (TC), increased serum levels of superoxide dismutase (SOD) and glutathione (GSH), and improved serum alcohol dehydrogenase (ADH) activity in the alcoholic fatty liver disease mice model. Cells and organisms rely on the Kelch-like ECH-associated protein 1- Nuclear factor erythroid 2-related factor 2 (Keap1-Nrf2) system as a critical defensive mechanism in response to oxidative stress. Therefore, Nrf2 plays an important role in ALD antioxidant responses, and its level is decreased by increased reactive oxidation stress (ROS) in the liver. ESF increased Nrf2, which was decreased in ethanol-damaged livers. Additionally, four polyphenol compounds were identified through a qualitative analysis of the ESF using LC-MS/MS. This study confirmed ESF’s antioxidative and hangover-elimination effects and suggested the possibility of using *Sophora flavescens* Aiton (SF) to treat ALD.

## 1. Introduction

Liver disease is often caused by hepatitis virus infection or drinking habits, such as excessive drinking, binge drinking, and frequent alcohol consumption [1,2]. Alcoholic liver disease (ALD) is the leading cause of steatosis progression to steatohepatitis, fibrosis, cirrhosis, and hepatocellular carcinoma and is responsible for 4% of global mortality [3,4,5]. Alcohol is mostly metabolized in the liver, but when it exceeds the liver’s capacity, liver function decreases, and various metabolic disorders are induced [3,6]. Long-term alcohol consumption causes impaired carbohydrate, protein, and lipid metabolism, leading to an excessive fatty acid supply [7]. In addition, alcohol induces an increase in fatty acid synthesis and damage to hepatocyte membranes as a result of impaired triglyceride secretion. This leads to alcoholic fatty liver disease, resulting in alcoholic hepatitis and liver cirrhosis [7,8]. Thus, early disease control, prior to the occurrence of steatohepatitis, could be of great significance in preventing the development of ALD [9]. Currently, despite the various treatments available for ALD, e.g., nutritional, pharmacological, psychotherapeutic, and surgical, the underlying mechanisms of alcohol-induced liver injury are unknown and unclear, and thus no satisfactory therapy is available [1,10,11]. However, anti-inflammation and antioxidant treatments are frequently used for ALD, such as silymarin and bifendate, which alleviate liver injury mainly by reducing free radical activities and the serum level of alanine aminotransferase (ALT) [12,13,14,15]. However, these treatments have limited clinical applications because of their side effects. Thus, finding effective treatments with fewer side effects, without compromising therapeutic effects, continues to be an important goal.

Recently, studies have focused on herbal medicines as materials for hangover removal as well as disease treatment [16,17]. *Sophora flavescens* Aiton (SF) is a plant species of the genus Sophora and the family Fabaceae, which is distributed throughout East Asia, mainly in China, Korea, and Japan [18,19]. SF is one of the oldest herbs used in traditional medicine. It helps regulate diuresis, remove toxicity and parasites, keep the heart clear, lessen dampness, and purge fire. [18,20]. According to a recent study, SF possesses a wide variety of biological activities, including anticancer, antibacterial, anti-inflammation, and antioxidant properties [20,21,22,23,24]. Rich polyphenols are reported to protect against the majority of chronic diseases [25] and the alkaloids and flavonoids in SF may have a crucial hepatoprotective role [26,27]. However, little is known about the mechanism of SF’s hepatoprotective effect in ALD treatment.

In the current study, we used the NIAAA mouse model, a single binge ethanol feeding in addition to chronic ethanol feeding for 10 days ad libitum oral feeding with the Lieber–DeCarli ethanol liquid diet to produce an established alcoholic liver damage in mice. The NIAAA model was developed at the NIAAA of the National Institutes of Health (NIH) by Dr. Bin Gao [28,29]. The NIAAA model has the advantage of being a low-cost, time-efficient, and easy-to-perform experimental animal model [29]. This model has a hallmark of hepatic neutrophil infiltration, elevation of serum ALT, AST, hepatocyte mitochondrial dysfunctions, and oxidative stress production [30]. This procedure method mimics acute-on-chronic ALD in patients by simultaneously inducing fatty liver, inflammation, high blood alcohol levels, and liver injury [29].

This study aimed to investigate the effect of SF ethanol extract on alcohol-induced injury in an NIAAA mouse model. In addition, indicators such as aspartate aminotransferase (AST), ALT, total cholesterol (TC), triglycerides (TG), glutathione (GSH), superoxide dismutase (SOD), and alcohol dehydrogenase (ADH) activity were assessed.

## 2. Materials and Methods

### 2.1. ESF Preparation

SF was purchased from the oriental herbal market in Yeongcheon (Yeongcheonsi, Gyeongsangbukdo, Republic of Korea) and authenticated by Professor Ki Hwan Bae, a medical botanist at Chungnam National University in the Republic of Korea. A total of 30 g of dried SF was ground into a powder, mixed with 390 mL of 70% ethanol, and then extracted via incubator shaking for 24 h. The extracts were then filtered through a testing sieve (150 μm, Retsch, Haan, Germany), evaporated, and concentrated through lyophilization. The obtained extract yield was 20.769%. The extract was named ESF. Then, ESF powder was transferred and sealed to the storage container and stored at −20 °C before use. For experiments, ESF powder was dissolved in distilled water (DW) (*v*/*v*). Dissolved ESF was filtered using a 0.22 μm disk filter.

### 2.2. Total Polyphenolic Content

The method was modified to identify the total phenolic content in ESF [31]. The total phenolic content was analyzed using the Folin–Ciocalteu reagent (Sigma-Aldrich, Sanit Louis, MO, USA). A total of 0.1 mL of ESF was mixed with 0.5 mL of Folin–Ciocalteu reagent, and 0.4 mL of a 7.5% sodium carbonate solution was added to the mixture. The absorbance at 750 nm was measured 1 h later in the dark at 20 °C. Gallic acid (Sigma-Aldrich, Saint Louis, MO, USA) was used for the standard curve. Gallic acid equivalents (GAEs mg/g) were used to express the total phenolic content of the sample.

### 2.3. Total Flavonoid Content

The method was modified to identify the total flavonoid content in ESF [31]. A colorimetric test was used to determine the total flavonoid concentration. A total of 860 μL of 80% EtOH was mixed with 100 μL of ESF. Then, 20 μL of 1M Potassium acetate (MENTOS Premade Solutions, Biostem, Suwon, Republic of Korea) was added, followed by 10% aluminum chloride (0.3 mL). The absorbance at 415 nm was measured 40 min later at 20 °C. Quercetin hydrate (Sigma-Aldrich, Saint Louis, MO, USA) was utilized as the standard for the calibration curve. The ESF total flavonoid content was expressed as QE mg/g, indicating milligram quercetin hydrate equivalents per gram of sample.

### 2.4. DPPH (2,2-Diphenyl-1-picrylhydrazyl) Assay

Using the DPPH (Thermo Scientific Fisher, Waltham, MA, USA) method, the antioxidant effect of ESF was investigated. The DPPH radical cation method was modified to value the free radical-scavenging effect [32]. Stock solutions of DPPH, in proper amounts, were mixed with methanol to produce assay solutions. DPPH stock solution with methanol yielded a usable mixture with an absorbance of around 1.00 at 517 nm. Ascorbic acid (AA, Sigma-Aldrich, Saint Louis, MO, USA) was used as a positive control, and the concentration was 100 μg/mL. ESF at various concentrations (10, 20, 40, 60, 80, 100, 200, and 300 μg/mL) and AA were added to 100 μL of DPPH solution, placed into a 96-well plate, and mixed thoroughly, and then the reaction was started. After that, the 96-well plate was incubated for 30 min at 37 °C. The absorbance was determined at 517 nm.

### 2.5. ABTS (2,2′-Azino-bis(3-ethylbenzothiazoline-6-sulfonic acid) Assay

The ABTS radical cation method was modified to evaluate the free radical-scavenging effect [32]. ABTS stock solutions were combined with 7.4 mM 2,2′-Azino-bis(3-ethylbenzothiazoline-6-sulfonic acid) and 2.6 mM potassium persulfate to create test solutions. The ABTS stock solution, prepared with DW, resulted in a combination with an absorbance of approximately 0.74 ± 0.02 at 734 nm. A total of 100 μg/mL AA was utilized as a positive control. Each concentration of 100 μL ESF (10, 20, 40, 60, 80, 100, 200, and 300 μg/mL) and AA were mixed with 100 μL of ABTS+ solution. The absorbance was measured at the maximum absorption wavelength of 734 nm.

### 2.6. Animals

C57BL/6 male mice, 6 weeks old, body weight (BW) 22 ± 2 g were purchased from Samtako Inc. (Osan, Republic of Korea). The mice were housed under a humidity of 50 ± 5%, a temperature of 23 ± 1 °C, a 12 h/12 h light and dark cycle, and were provided free access to a standard diet and tap water. All mice were provided with proper care following the guidelines of the Institutional Animal Care and Use Committee (IACUC) of the Korea Institute of Oriental Medicine (KIOM; Daegu, Republic of Korea), who reviewed and approved this study (IACUC-KIOM-D-18-013).

### 2.7. Diets, Treatments, and Sample Collection

**Figure 1:**
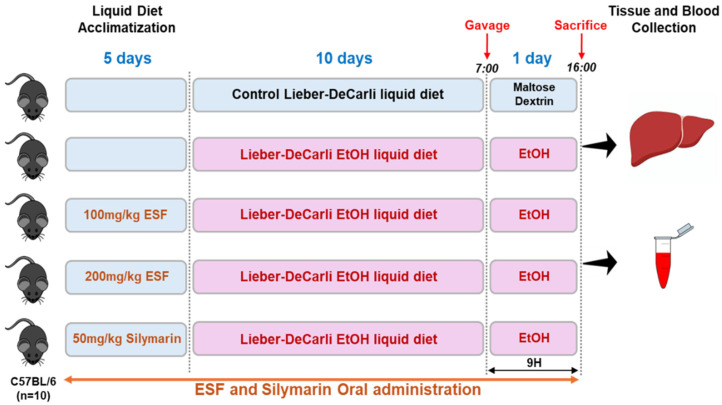
Basic overview of the model procedures (National Institute on Alcohol Abuse and Alcoholism (NIAAA) model).

Many previous studies used eight mice per group to assess the differences in metabolites, proteins, and biochemicals between them [33,34]. In this study, mice were randomly separated into five groups with ten mice per group. Thus, the number of samples in this study was likely to result in sufficient power, even if power analysis was not performed. We carried out the experimental procedure in accordance with the NIAAA model [28,29]. An overview of the experimental design is outlined in Figure 1. On days 1–5, all mice had free access to the control Lieber–DeCarli diet (#710027, Dyets, Inc., Bethlehem, PA, USA) for acclimatization to a liquid diet. From day 6, the ethanol-alone group, ESF-treated group (ESF, 100 and 200 mg/kg/BW), and positive control group (silymarin, 50 mg/kg/BW) were allowed free access to the 5%-ethanol-containing Lieber–DeCarli diet (#710260, Dyets, Inc., Bethlehem, PA, USA) for 10 days. The control group was fed the control Lieber–DeCarli diet. On day 16, ethanol-fed mice and the control group were orally administered a single dose of ethanol (5 g/kg/BW) or maltose dextrin (9 g/kg/BW) at 7 am, respectively. After 9 h, mice were euthanized, and liver tissues and blood were collected. During the experimental period, ESF and silymarin were orally administered at the same time every day, and dietary intake and BW were measured at the same time every day.

### 2.8. Histological Analysis

We removed the liver tissue of the largest right lobe, fixed it in 10% formalin in DW, embedded it in paraffin, cut it into 5 μm, and stained it with hematoxylin and eosin (H&E), according to our previous report [35]. Then, phase-contrast microscopy was used to examine H&E-stained liver sections.

### 2.9. Biochemical Assays

Blood samples obtained from mice were separated by centrifugation at 12,000 rpm for 10 min and obtained serum. The serum was stored at −80 °C before use [36]. AST or ALT assay kits (AST; ab105135, ALT; ab105134, Abcam, Cambridge, UK) were used to measure the activities of serum ALT and AST in order to estimate alcohol-induced liver injury. The activity of SOD and the levels of GSH were determined using a SOD assay kit or a GSH assay kit (SOD; 706002, GSH; 703002, Cayman, Ann Arbor, MI, USA) to assess alcohol-induced oxidative stress in the serum. Serum triglyceride (TG) and cholesterol levels were detected by using triglyceride (TG) and cholesterol assay kits (TG; ab65336, cholesterol; ab65390, Abcam, Cambridge, UK). ADH activity was analyzed using a colorimetric assay kit (ab102533, Abcam, Cambridge, UK). Each biochemical analysis was performed according to the manufacturer’s instructions in the kit.

### 2.10. Protein Extraction and Western Blot Analysis

The protein extraction and Western blot analysis methods were modified and performed [37]. To obtain the total protein from tissues, a radioimmunoprecipitation assay (RIPA) buffer (Thermo Scientific Fisher, Waltham, MA, USA) was added to an Xpert duo inhibitor cocktail solution (GenDEPOT, Katy, TX, USA) and then lysed and extracted. Protein quantification was measured using a BCA reagent (BIOMAX, Guri, Republic of Korea). Extracted proteins were mixed with 5× sample buffer (Cat#EBA-1052, ELPiS, Daejeon, Korea) and heated at 80 °C for 15 min. Then, 20 μg of protein was separated using sodium dodecyl sulfate-polyacrylamide (SDS) gel electrophoresis and transferred to polyvinylidene difluoride (PVDF) membranes (Millipore, Burlington, MA, USA). The membranes were blocked in 5% skimmed milk for 2 h at room temperature and washed with Tris-buffered saline, containing 1% Tween 20 (TBST) buffer, for 30 min. Specific primary antibodies, Nrf2 (Cat#ab137550, Abcam, Cambridge, UK), and β-actin (Cat#ATGA0570, NKMAX, Seongnam, Republic of Korea) were diluted 1:1000 in TBST buffer and added to the membranes. Then, this mixture was incubated on a rocker overnight at 4 °C. After washing with TBST buffer, the membranes were incubated with anti-mouse or anti-rabbit secondary antibody (diluted 1:5000) for 2 h at 20 °C. The resulting immunoblots were detected using SuperSignal™ West Pico PLUS Chemiluminescent Substrate (Cat# 34580, Thermo Scientific Fisher, Waltham, MA, USA) and an imaging system (SH-Compact 523+, Shenhua Science Technology, Hangzhou, China), according to the manufacturer’s instructions.

### 2.11. Liquid Chromatography-Tandem Mass Spectrometry (LC-MS/MS) Conditions for ESF

LC and LC-MS/MS methods were modified and performed [38]. LC and LC-MS/MS were performed on a 1260 series LC system (Agilent Technologies, Inc., Santa Clara, CA, USA), and a 3200 QTrap tandem mass system (Sciex LLC, Framingham, MA, USA) was operated in negative ion mode (spray voltage set at −4.5 kV). The solvent used was DW (solution A) and 0.1% formic acid contained Acetonitrile (solution B). For analysis, a gradient system was set flow rate at 0.5 mL/min, and a prontosil C18 column (length, 250 mm; inner diameter, 4.6 mm; particle size, 5 µm; Bischoff Chromatography, Phenomenex Co., Ltd., Torrance, CA, USA) was used. The solvent conditions used in the mobile phases were 0–10 min at 10–15% solution B, 10–20 min at 20% solution B, 20–30 min at 25% solution B, 30–40 min at 40% solution B, 40–50 min at 70% solution B, 50–60 min at 95% solution B, and 60–70 min at 95% solution B. The analysis was measured at 284 nm at 35 °C.

### 2.12. Molecular Docking Analysis for ESF

Molecular docking is a powerful technique for research of receptor–ligand interaction inhibition of enzymes related to the antioxidant activity of compounds [39]. It was performed to analyze the possible binding mode of the Keap1 ligand and the ability of Epigallocatechin, Isoxanthohumol, Kurarinone, and Kushenol F to inhibit interaction. Experimentally determined protein structures from the RCSB Protein Data Bank (RCSB PDB) were retrieved to perform a molecular docking analysis (https://www.rcsb.org/, accessed on 20 August 2023) using the search Kelch domain of human Kelch-like ECH-associated protein 1 (Keap1, ID: 1U6D). The 3D compound structures of Epigallocatechin (Compound CID: 72277), Isoxanthohumol (Compound CID: 513197), Kurarinone (Compound CID: 11982640), Kushenol F (Compound CID: 72936), and CPUY192018 (Compound CID: 73330369) were searched and then downloaded from PubChem (https://puchem.ncbi.nlm.nih.gov/, accessed on 5 October 2023). Docking analysis was carried out using UCSF Chimera 1.17.3 and Auto-Dock module Vina with default settings [40]. The results of docking were visualized using Discovery Studio software 4.0 [41]. The binding affinities were calculated based on estimated free energy binding and total intermolecular energy. All experiments were performed in triplicate and at a root-mean-square deviation (RSMD) of ≤2 Å.

### 2.13. Statistical Analysis

All statistical analyses were performed using GraphPad Prism 8.0 (GraphPad Software, Inc., San Diego, CA, USA). The data are expressed as the mean ± standard error of the mean (SEM), and Brown–Forsythe and Welch analysis of variance (ANOVA) with Dunnett T3 multiple comparison tests were then conducted, and a *p*-value < 0.01 was regarded as statistically significant. Additionally, one-way and two-way factorial ANOVA were used to determine whether there were significant differences between the groups. Tukey’s and multiple comparison tests were then conducted, and a *p*-value < 0.05 was regarded as statistically significant.

## 3. Results

### 3.1. The Total Phenolic and Flavonoid Content of the ESF

The total phenolic content of the ESF was estimated using gallic acid and expressed as mg gallic acid equivalent (GAE)/g of extract. Additionally, the total flavonoid content of the ESF was expressed as mg quercetin equivalents (QE)/g of extract. The total polyphenol content was determined to be 55.076 ± 0.77 mg GAE/g, and the total flavonoid content was measured at 55.876 ± 1.211 mg QE/g. Table 1 represents the analytical data for the total phenolic and flavonoid content of the ESF.

### 3.2. Effects of ESF on Antioxidant Activity

**Figure 2:**
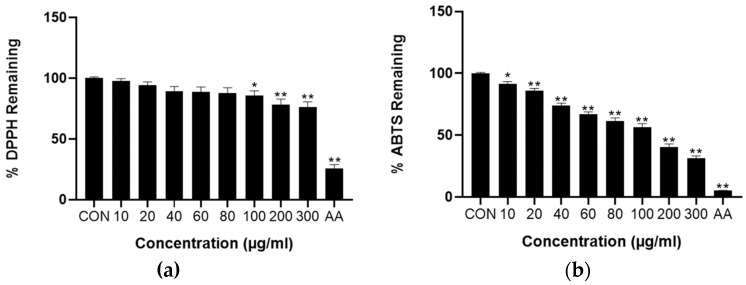
Antioxidant capacities measured at various concentrations of ESF. (**a**) Percentage of DPPH remaining of ESF. (**b**) Percentage of ABTS remaining of ESF. Data are shown as the mean ± SEM (*n* = 20). Statistical analyses were different values according to the Brown–Forsythe and Welch ANOVA with Dunnett T3 (Control vs. ESF or AA, ** *p* < 0.001, * *p* < 0.01). CON: control, AA: ascorbic acid.

In this study, the DPPH and ABTS assays were performed to evaluate the free radical-scavenging capacity of ESF. The results indicated that ESF had the ability to scavenge DPPH and ABTS in a concentration-dependent manner. As shown in Figure 2a, the DPPH radical remained and ESF decreased with an increase in its concentration, showing a range of 7.5–19.15%. On the other hand, ESF demonstrated the ability to eliminate ABTS·+ radicals, exhibiting a biphasic concentration-dependent activity of approximately 53.4%, starting from 60 μg/mL (Figure 2b). Through DPPH and ABTS analysis, it was determined that SF has an excellent free radical-scavenging ability. This means it can prevent the destruction of antioxidant balance and maintain metabolic homeostasis in the liver.

### 3.3. Effects of ESF on Dietary and Body Weight (BW)

After feeding them alcohol for 10 days, the dietary intake and weight gain were measured in mice. The amount of dietary intake was 11.5 ± 0.7 mL/day in mice fed only the normal diet. However, mice fed with an alcohol diet (9.4 ± 0.4 mL/day) were reduced, but it was not significant (Table 2). The BW was 28.19 ± 0.47 g in the mice fed only the normal diet, and there was little difference between the mice fed only the alcohol diet (24.90 ± 0.31 g) significantly reduced (Table 2). In the case of the mice fed with ESF and an alcohol diet, the weight gain rate was not significantly different from that of the mice fed only the alcohol diet (Table 2).

### 3.4. Effects of ESF on Alcohol-Induced Liver Injury

**Figure 3:**
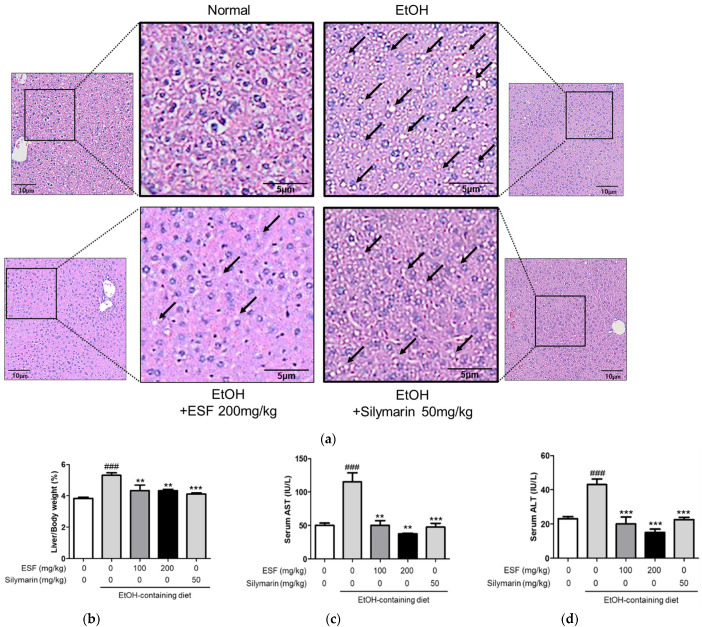
Effects of ESF on ethanol-induced liver injury. (**a**) H&E-stained liver sections of EtOH-induced liver injury model. The droplets are indicated by the black arrow. All images are visualized at 100×, (*n* = 10). (**b**) Liver/BW (%). (**c**) Activities of AST in serum. (**d**) Activities of ALT in serum. Data are shown as the mean ± SEM (*n* = 10). Statistical analyses were different values according to the one-way ANOVA with Tukey’s range test (Normal diet vs. EtOH diet ### *p* < 0.001; EtOH diet vs. EtOH diet + ESF or silymarin *** *p* < 0.001, ** *p* < 0.01).

ESF administration was confirmed to be hepatoprotective against alcohol-induced liver damage. As a result of measuring the liver weight per BW, the mice fed with an alcohol diet significantly increased, and the mice fed with the alcohol diet supplementary with ESF significantly decreased compared to those fed with only the alcohol diet (Figure 3b). In addition, as a result of H&E staining to confirm the hepatoprotective effect histologically, a large number of droplets (black arrows) were observed overall in ethanol-induced liver tissue. These droplets were significantly suppressed in mice administered ESF (Figure 3a). Also, we measured the serum AST and ALT activity, indicating liver function and degree of damage [42]. In our study, serum AST and ALT activity was significantly increased by alcohol diet administration, and AST and ALT activity increase due to alcohol was decreased the administered with ESF in a dose-dependent manner, suggesting that ESF helps ameliorate ethanol-induced liver injury (Figure 3c,d).

### 3.5. Effects of ESF on Triacylglycerol (TG) and Total Cholesterol (TC) Levels

**Figure 4:**
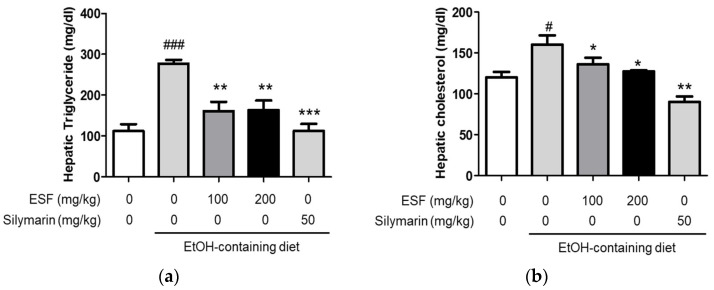
Effects of ESF on alcoholic fatty liver disease. (**a**) Hepatic triacylglycerol levels. (**b**) Hepatic cholesterol levels. Data are shown as the mean ± SEM (*n* = 10). Statistical analyses were different values according to the one-way ANOVA with Tukey’s range test (Normal diet vs. EtOH diet ### *p* < 0.001, # *p* < 0.05; EtOH diet vs. EtOH diet + ESF or silymarin *** *p* < 0.001, ** *p* < 0.01, * *p* < 0.05).

TG and TC are important indicators of fat accumulation by alcohol consumption in liver injury [43]. Therefore, we confirmed the effect of the ESF administration on alcohol-derived TG and TC levels. Our results showed that the mice fed with an alcohol diet obviously increased both serum TG and TC levels, and the mice fed with the alcohol diet supplementary with ESF and silymarin remarkably decreased compared to those fed with only the alcohol diet (Figure 4). These results indicated that ESF pretreatment also effectively recovered the ethanol-induced liver dysfunction compared with silymarin as a positive control.

### 3.6. Effects of ESF on Hepatic Antioxidant and Oxidative Stress Markers

**Figure 5:**
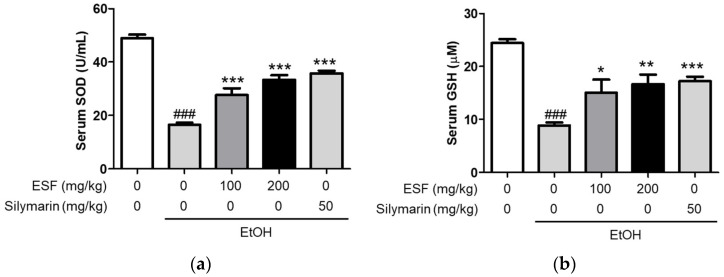
Effects of ESF on liver antioxidant defense. (**a**) Activities of serum SOD. (**b**) Activities of serum GSH. Data are shown as the mean ± SEM (*n* = 10). Statistical analyses were different values according to the one-way ANOVA with Tukey’s range test (Normal diet vs. EtOH diet ### *p* < 0.001; EtOH diet vs. EtOH diet + ESF or silymarin *** *p* < 0.001, ** *p* < 0.01, * *p* < 0.05).

To evaluate the effect of ESF on the antioxidant effects, we confirmed the serum SOD and GSH levels [44]. The mice fed with an alcohol diet significantly decreased both serum SOD and GSH levels, and the mice fed with the alcohol diet supplementary with ESF and silymarin considerably recovered compared to those fed with only the alcohol diet (Figure 5).

### 3.7. Effects of ESF on Alcohol Dehydrogenase (ADH) Activity

**Figure 6:**
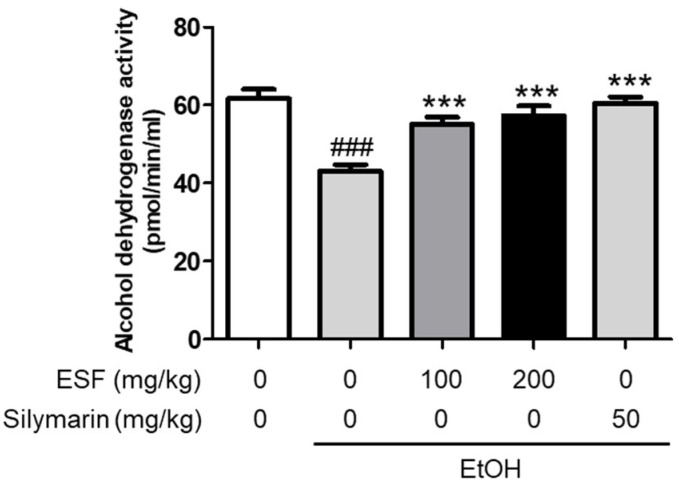
Effects of ESF on ADH. Data are shown as the mean ± SEM (*n* = 10). Statistical analyses were different values according to the one-way ANOVA with Tukey’s range test (Normal diet vs. EtOH diet ### *p* < 0.001; EtOH diet vs. EtOH diet + ESF or silymarin *** *p* < 0.001).

As a result of confirming the effect of ESF administration on ADH activity [45], serum ADH activity was significantly reduced in mice fed with an alcohol diet, and ADH activity decreased by alcohol was significantly increased by ESF and silymarin (Figure 6).

### 3.8. Western Blotting

**Figure 7:**
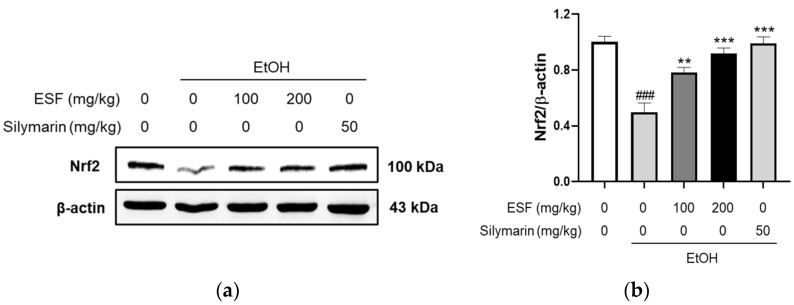
Effects of ESF on Western blot. (**a**) Nrf2 protein expression showed the effect of 100 or 200 mg/kg ESF and 50 mg/kg silymarin. (**b**) Nrf2 expression level was normalized by β-actin. Data are shown as the mean ± SEM (*n* = 10). Statistical analyses showed different values according to the one-way ANOVA with Tukey’s range test (Normal diet vs. EtOH diet ### *p* < 0.001; EtOH diet vs. EtOH diet + ESF or silymarin *** *p* < 0.001, ** *p* < 0.01).

Nuclear factor erythroid 2-related factor 2 (Nrf2) regulates reactive oxidation stress (ROS) production. The enormous accumulation of ROS affects cell function, which could trigger cell death. Nrf2 activates to resist oxidation by ROS. To evaluate the effect of ESF on the Nrf2 activation effects, we confirmed the Nrf2 expression through Western blot. The mice fed with an alcohol diet significantly decreased Nrf2 expression, and the mice fed with the alcohol diet supplementary with 100 and 200 mg/kg ESF and 50 mg/kg silymarin considerably increased to those fed with only the alcohol diet. In particular, Nrf2 expression of 200 mg/kg ESP was similar to 50 mg/kg silymarin. This result means that ESF protects the injured liver through the EtOH diet (Figure 7).

### 3.9. Qualitative Determination of ESF by LC-MS/MS

**Figure 8:**
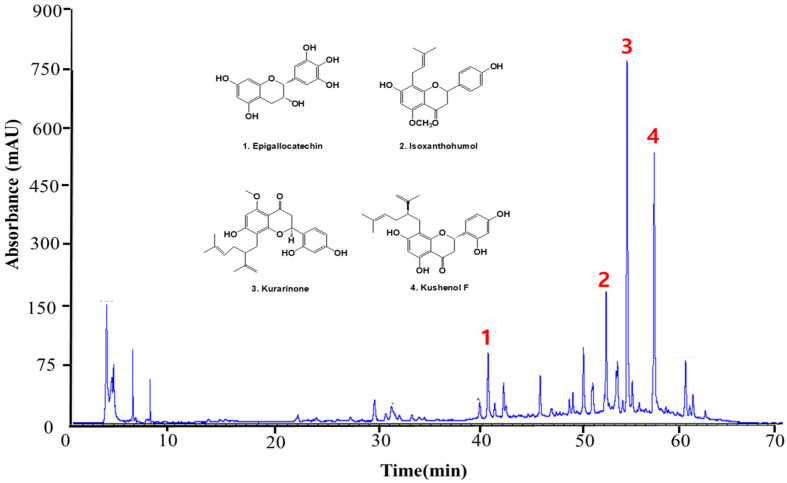
HPLC chromatogram and structure of the polyphenolic compounds in ESF. Epigallocatechin (1), Isoxanthohumol (2), Kurarinone (3), and Kushenol F (4) were the detected compounds at 284 nm.

**Figure 9:**
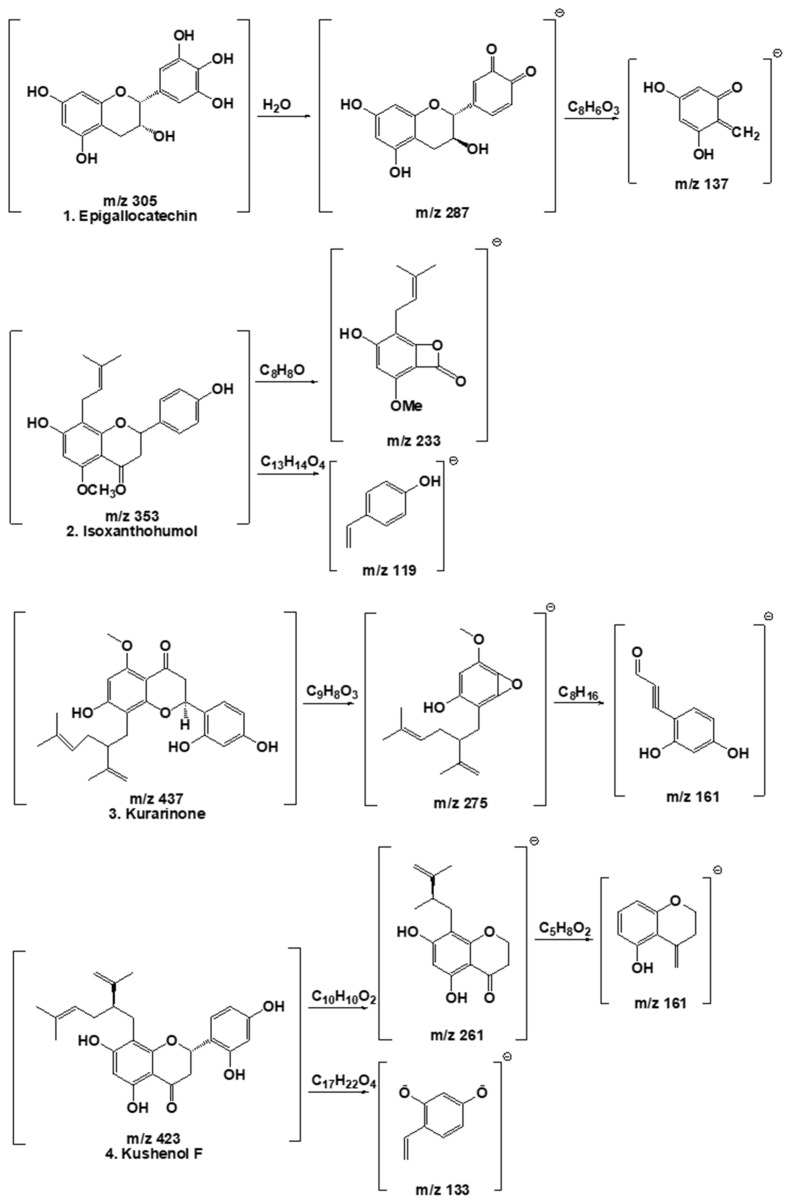
Fragmentation scheme of compounds contained in ESF.

A qualitative analysis of the compounds contained in the ESF was identified by LC-MS/MS. A total of four peaks were identified based on LC retention time and ultraviolet (UV)-vis spectrum (Figure 8). Phenolic compounds were identified as Epigallocatechin (1) [46], Isoxanthohumol (2) [47], Kurarinone (3) [48], and Kushenol F (4) [48]. Four phenolic compounds were identified according to the peaks obtained via high-performance liquid chromatography (HPLC) at a wavelength of 248 nm. The tentative identification of the extract components was based on molecular weights, MS/MS fragmentation, as well as literature data. Compounds were numbered according to their elution order (retention times) (Table 3). Peaks 1, 2, 3, and 4 showed precursor ions [M−H]^−^ at m/z 304.074 (C15H14O7), 439.204 (C21H22O5), 354.147 (C26H30O6), and 423.189 (C26H32O7), respectively. The formation of these compound fragments is presented in Figure 9.

### 3.10. Molecular Docking Analysis of Compounds with Keap1 from ESF

**Figure 10:**
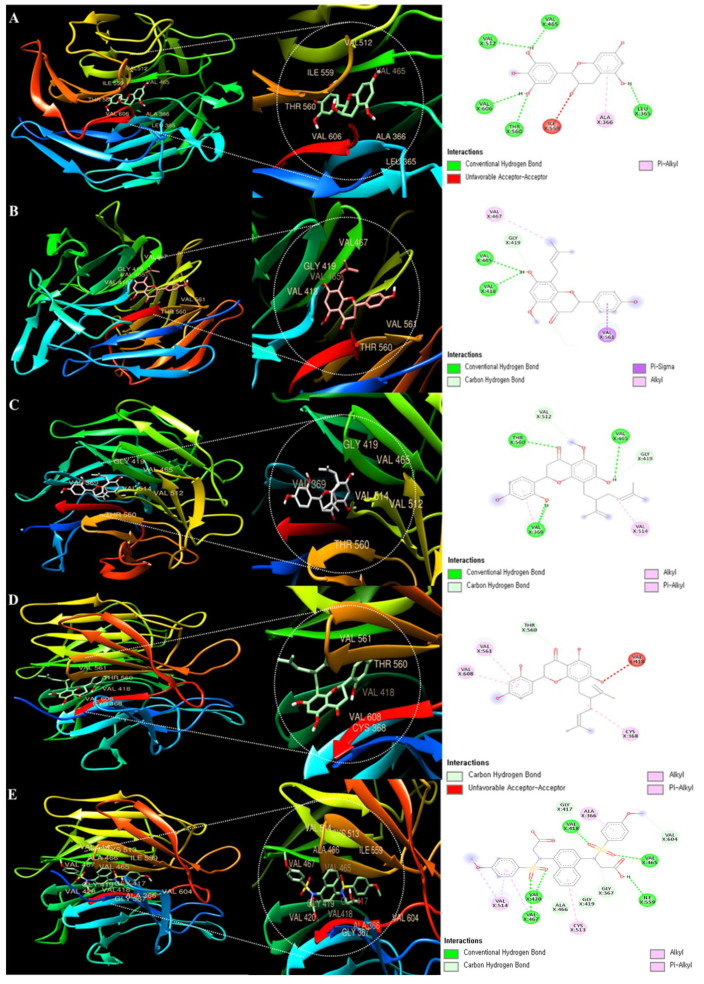
Molecular docking analysis of ESF. (**A**) Epigallocatechin; (**B**) Isoxanthohumol; (**C**) Kurarinone; (**D**) Kushenol F; and (**E**) CPUY192018. These were bound to the Keap1 3D structure. Each color represents a direct-bond type of interaction. CPUY192018: positive control.

Keap1 functions as a substrate adaptor protein for a ubiquitin ligase complex, which specifically targets the Nrf2 transcription factor for destruction [49]. Therefore, molecular docking was performed with Keap1 using four polyphenol compounds (Epigallocatechin, Isoxanthohumol, Kurarinone, and Kushenol F) identified in Figure 8. Additionally, Table 4 shows the docking scores, demonstrating how each protein and ligand bind to the complementary surface (Figure 10) and how much energy it takes for this to occur.

Ligand/protein docking was assessed using the UCSF Chimera program. Table 3 shows that the following active sites of Epigallocatechin were bound to Keap1: VAL 465, LEU 365, ALA 366, ILE 559, THR 560, VAL 606, and VAL 512. The molecular binding energy score of syringin was determined to be −9.2 kcal/mol. The molecular docking of Isoxanthohumol to Keap1 revealed the following active sites: VAL 465, GLY 419, VAL 467, THR 560, VAL 561, and VAL 418, with a molecular binding energy score of −6.9 kcal/mol. Kurarinone promoted Keap1 binding to the most abundant active sites (VLA 465, GLY 419, VAL 514, VAL 369, THR 560, VAL 512).

The molecular binding energy score of Kurarinone was found to be −7.9 kcal/mol. Kushenol F promoted Keap1 binding to the most abundant active sites (VAL 465, ALA 607, VAL 606, ILE 559, ALA 366, GLY 364, ILE 416, ARG 415, ALA 556, GLY 417, VAL 418), and the docking also showed the highest molecular binding energy score of −7.9 kcal/mol. The molecular docking of CPUY192018 to Keap1 revealed the following active sites: GLY 417, VAL 418, ALA 366, VAL 604, VAL 465, ILE 559, GLY 367, GLY 419, CYS 513, ALA 466, VAL 420, VAL 467, and VAL 514, with a molecular binding energy score of −9.0 kcal/mol. The active site of Kushenol F on Keap1 was higher than that of the positive control group, CPUY192018.

## 4. Discussion

The liver is a major detoxification organ [50], and it plays a crucial role in maintaining metabolic homeostasis [51]. Additionally, the liver metabolizes various compounds that generate free radicals. The disruption of liver metabolic homeostasis due to the disruption of oxidative and antioxidant balance causes oxidative stress [52]. Eventually, this will lead to liver disease. Therefore, the recovery of antioxidants is essential as they have free radical-scavenging abilities and thus can maintain liver homeostasis. An excellent antioxidant effect was observed through the free radical scavenging of ESF (Figure 2).

To establish a liver pathology similar to clinical ALD, rodents were orally administered ethanol, producing an effective and accurate animal model [28,29]. In this study, ESF and silymarin concentrations were established with antioxidants of ESF results and reference to previous studies [26,53,54,55]. Our present study showed that alcohol intake for 10 days significantly increased serum ALT, AST (Figure 3c,d), TG, and TC levels (Figure 4). Food consumption was decreased in the group that consumed alcohol compared to the group that consumed the regular diet, but it was not significant (Table 2). In this study, the weight of the mice decreased after alcohol treatment, and there was no significant change in BW following the administration of ESF (Table 2 and Figure 3b). ALT and AST are important metabolic enzymes in liver cells and generally have low plasma levels. Therefore, serum ALT and AST are usually considered to be optimal markers for diagnosing liver injury [56,57]. In this study, significant increases in ALT and AST were observed after alcohol administration (Figure 3c,d), indicating that alcohol treatment can damage plasma and hepatocytes. ESF pretreatment attenuated the elevation of ALT and AST at a concentration of 200 mg/kg (Figure 3c,d).

Previous studies have shown that fat accumulation occurs through a common and complex process in liver cells, resulting from the ingestion of alcohol [58,59]. In this study, serum triglyceride and cholesterol contents in the alcohol group were significantly increased, compared to those in the normal control group, and were significantly reduced following ESF pretreatment (Figure 4). When liver tissue was observed through H&E staining, pathological lesions were recovered due to alcohol-induced treatment damage (100 and 200 mg/kg) (Figure 3a). Excessive alcohol intake induces oxidative stress, impairing antioxidant defenses and simultaneously generating reactive oxygen species [45,60,61,62]. Alcohol-induced oxidative damage was detected by measuring the serum SOD and GSH contents. Alcohol treatment resulted in 2.9-fold lower SOD levels and 2.7-fold lower GSH levels in serum compared to controls. In contrast, it was confirmed that concentration-dependent recovery was achieved when ESF was pre-treated (Figure 5).

Finally, the activity of ADH was measured to confirm the effect of ESF on liver alcohol metabolism [63]. It was confirmed that the activity of ADH, which had been decreased by alcohol, was increased by the administration of ESF (Figure 6). These results indicate that ESF treatment improves both antioxidant effects as well as alcohol-induced hangovers.

To further study the potential effects of antioxidative stress and ESF oxidant defense mechanisms on alcoholic liver injury, Nrf2 expression was evaluated in liver tissues. Nrf2 has a crucial protective function in alcohol-induced hepatic damage, which is mainly related to its principal role in the antioxidant response to ethanol metabolism [64]. In normal conditions, Nrf2 activity is suppressed by binding to the repressor Keap1 [65]. On the other hand, under oxidative stress states, Nrf2 dissociates from Keap1, moves into the nucleus, and combines with transcription factors [65,66]. After this, the complex associates with the antioxidant response element (ARE) and accelerates the transcription of antioxidant genes such as heme oxygenase-1 (HO-1) and GSH [66]. Therefore, Nrf2 has a protective role against ethanol-induced hepatic damage and is important in ALD therapy [67]. In our study, ESF increased Nrf2, which was decreased in the liver tissue damaged by an ethanol diet (Figure 7). This suggests that ESF alleviates liver damage by regulating Nrf2.

Polyphenol is a secondary metabolite found in many plants and is a general term for compounds with multiple phenolic hydroxyl groups in a molecule [68]. Polyphenols have recently gained attention as functional foods and pharmaceutical raw materials due to their physiological activities, such as arteriosclerosis and hypertension prevention, and hepatoprotective, anti-inflammatory, and antitumor activity [69]. Polyphenols are divided into flavonoids, xanthones, coumarins, and phloroglucins, with over 7000 flavonoids found in nature. Plants biosynthesize polyphenols that are prenylated, methylated, and glycosided, and the physiological activities of approximately 1000 polyphenols with a prenyl group have been discovered [70]. Overall, four types of ESF compounds were identified as a result of LC-MS/MS analysis (Figure 8 and Figure 9 and Table 3). Isoxanthohumol [46] is a prenylflavonoid, while Epigallocatechin [47], Kurarinone [48], and Kushenol F [48] are polyphenolic compounds. Furthermore, Kushenol F is a standard SF material. Based on previous research, this study looked at the change in relative peak area before and after the reaction. It was based on the idea that each peak demonstrates the compound’s reactivity and that these reactions could have an antioxidant effect [69]. Kushenol F and Epigallocatechin exhibited significant binding activity (Figure 10 and Table 4). These findings indicate that the polyphenolic chemicals present in the extract possess anti-inflammatory properties, not only concerning their potential antioxidant activities but also in terms of their affinity for ligand/protein structures. Moreover, the extract’s putative anti-inflammatory action is reinforced by both the expected docking results and the in vitro results.

Recent work has revealed that cytochrome P450 2E1 enzymes induced by chronic alcohol exposure can result in producing excessive ROS, which is related to lipid peroxidation. SF has potential due to its various polyphenol and flavonoid constituents that have effects on suppressing ROS and NO production [71]. Among SF compounds, Epigallocatechin and Kurarinone have a significant antioxidant capacity [72,73], and Kushenol F and Isoxanthohumol decreased the phosphorylation of NF-κB and the levels of the proinflammatory cytokines [74,75]. It suggests that ESF is related to anti-inflammatory properties in alcoholic liver disease. This point means SF could alleviate inflammation in ethanol-induced mouse liver. It proved ESF ameliorates liver injury in the NIAAA model in this study. 

The antioxidant and hepatoprotective effects of ESF’s polyphenolic compounds were considered, but further research is needed. A limitation of this study is that it did not investigate the role of critical inflammatory mediators, such as IL-6, TNF-α, and IL-1β, in the hepatoprotective mechanism mediated by ESF’s antioxidant effect using the NIAAA model. These mediators will be examined in further research.

## 5. Conclusions

In conclusion, we investigated the hepatoprotective effect of oral ESF administration on alcohol-induced hepatotoxicity using a mouse model. After inducing hepatotoxicity through an alcohol diet, liver tissue shape, liver function indicators (ALT, AST), serum TG, TC, SOD, and GSH contents, and ADH activity were investigated. Oral ESF administration inhibited fat accumulation in the liver tissue, due to the alcohol-induced hepatotoxic state, improved serum AST and ALT levels, and increased the serum concentrations of the antioxidants SOD and GSH. In addition, hepatic TG and TC contents, which are related to fat accumulation, were also reduced, and ADH activity was increased due to hangover improvement. Additionally, ESF inhibited the alcohol-induced decrease in Nrf2, demonstrating hepatoprotection. Therefore, this suggests that ESF has potential hepatoprotective effects against alcohol-induced oxidative stress and hangover reactions.

## Figures and Tables

**Table 1:** Total phenolic and flavonoid content of ESF.

Items	Concentration
Total polyphenolic content (GAE mg/g) ^1^	55.076 ± 0.77
Total flavonoid content (QE mg/g) ^2^	55.876 ± 1.211

^1^ Total polyphenolic content is expressed as gallic acid equivalents. ^2^ Total flavonoid content is expressed as quercetin equivalents.

**Table 2:** Effect of ESF on food intake and body weight (BW) changes.

Groups	Food Intake (ml/Day)		BW (g)	
	5 Day	15 Day	Initial	Final
Normal Diet	12.1 ± 1.1	11.5 ± 0.7	22.43 ± 1.00	28.19 ± 0.47
EtOH Diet	12.7 ± 1.1	9.4 ± 0.4	22.73 ± 0.45	24.90 ± 0.31 ##
EtOH Diet + ESF (100 mg/kg)	11.7 ± 1.4	9.9 ± 0.1	22.32 ± 1.23	25.35 ± 0.22
EtOH Diet + ESF (200 mg/kg)	11.2 ± 1.8	9.8 ± 0.3	23.62 ± 0.31	24.98 ± 0.30
EtOH Diet + Silymarin (50 mg/kg)	11.2 ± 1.8	8.8 ± 0.3	23.33 ± 0.31	24.34 ± 0.44

Data are expressed as mean ± SEM (*n* = 10). Statistical analyses were different values according to the two-way ANOVA with Tukey’s range test (Normal diet vs. EtOH diet ## *p* < 0.01).

**Table 3:** The LC-MS/MS data for the polyphenolic compounds in ESF.

Peak No.	^1^ Rt (Min)	Formula	Compound	[M-H]^−^	MS/MS	References
1	40.62	C15H14O7	Epigallocatechin	305	287, 137	[46]
2	52.16	C21H22O5	Isoxanthohumol	353	233, 119	[47]
3	54.20	C26H30O6	Kurarinone	439	275, 161	[48]
4	56.86	C26H32O7	Kushenol F	423	261, 161, 133	[48]

^1^ Rt: Retention time.

**Table 4:** Molecular docking studies and binding energy (1U6D) of compounds in ESF bound to Keap1.

Binding Ligand	Amino Acid Residue that Interacts	Docking Score
Epigallocatechin	VAL 465, LEU 365, ALA 366, ILE 559, THR 560, VAL 606, VAL 512	−9.2 kcal/mol
Isoxanthohumol	VAL 465, GLY 419, VAL 467, THR 560, VAL 561, VAL 418	−7.5 kcal/mol
Kurarinone	VLA 465, GLY 419, VAL 514, VAL 369, THR 560, VAL 512	−7.9 kcal/mol
Kushenol F	VAL 465, ALA 607, VAL 606, ILE 559, ALA 366, GLY 364, ILE 416, ARG 415, ALA 556, GLY 417, VAL 418	−10.3 kcal/mol
CPUY192018	GLY 417, VAL 418, ALA 366, VAL 604, VAL 465, ILE 559, GLY 367, GLY 419, CYS 513, ALA 466, VAL 420, VAL 467, VAL 514	−9.0 kcal/mol

## Data Availability

Data are contained within the article.
